# Determinants of cancer care delays in Kinshasa, Democratic Republic of the Congo (DRC)

**DOI:** 10.64898/2026.05.19.26353550

**Published:** 2026-05-26

**Authors:** Jean Claude Dusingize, Natalia Zotova, Rafi Kabarriti, Kavita Sehrawat, Pélagie Babakazo, Ali Shongo Alisho, Fidele Lumande Kasindi, Ilyass Yessoufou, Marcel Yotebieng

## Abstract

**PURPOSE:**

Cancer outcomes in sub-Saharan Africa are driven by delayed diagnosis and treatment initiation. We evaluated the magnitude and determinants of diagnostic and treatment delays among cancer patients in Kinshasa, Democratic Republic of the Congo (DRC).

**METHODS:**

We conducted a hospital-based cross-sectional study of 460 adults with confirmed cancer at Nganda Hospital Center in Kinshasa, DRC. Two outcomes were assessed: delay from symptom onset to diagnosis and delay from diagnosis to treatment initiation. Log-normal regression models were fitted for each outcome to estimate adjusted geometric mean ratios (aGMRs) and 95% confidence intervals (CIs). Covariates included demographic, socioeconomic, clinical, behavioral, and stigma-related factors.

**RESULTS:**

The median age was 55 years, and 76.2% of participants were women. Overall, 55.0% of participants experienced symptom-to-diagnosis delays >6 months, and 49.4% experienced diagnosis-to-treatment delays >3 months. Older age was associated with longer diagnostic delay (aGMR 1.55, 95% CI 1.03-2.31) and treatment delay (1.51, 1.07-2.14). Unemployment was strongly associated with both diagnostic delay (1.68, 1.15-2.47) and treatment delay (2.27, 1.54-3.33), as was hepatitis B co-infection (1.88, 1.06-3.34 and 2.42, 1.15-5.11, respectively). Longer diagnostic delay was additionally associated with informal trading (1.99, 1.21-3.28), taxi or motorbike transport (1.92, 1.25-2.94), and smoking history (2.25, 1.03-4.91), while high cancer-stereotype stigma was associated with longer treatment delay (1.56, 1.04-2.34).

**CONCLUSION:**

Substantial delays exist across the DRC cancer care continuum, driven by socioeconomic vulnerability, transport barriers, hepatitis B co-infection, and cancer-related stigma. These findings highlight the need for integrated interventions to improve timely diagnosis and treatment initiation, including strengthening financial protection, decentralizing cancer services, and reducing stigma in cancer care.

## Full Text Availability

The license terms selected by the author(s) for this preprint version do not permit archiving in PMC. The full text is available from the preprint server.

